# Division of coal spontaneous combustion stages and selection of indicator gases

**DOI:** 10.1371/journal.pone.0267479

**Published:** 2022-04-27

**Authors:** Zongxiang Li, Mingqian Zhang, Zhibin Yang, Jingxiao Yu, Yu Liu, Haiwen Wang

**Affiliations:** 1 College of Safety Science and Engineering, Liaoning Technical University, Fuxin, Liaoning, China; 2 Key Laboratory of Mine Thermodynamic Disaster & Control of Ministry of Education, Liaoning Technical University, Huludao, Liaoning, China; Minia University, EGYPT

## Abstract

Investigating the division of coal spontaneous combustion stages and the selection of indicator gases is significant to the safe production of coal mines. In this study, the characteristic temperature of coal spontaneous combustion, the generation law of indicator gases, the combustion process, and the division of the combustion stages of coal samples taken from Hongqingliang (HQL) and Dayan (DY) mines were investigated using thermogravimetric analysis experiment, indicator gas detection experiment, and coal oxidation spontaneous combustion experiment. The results of the thermogravimetric analysis experiment showed that the pyrolysis temperatures of the HQL and DY coals were 115.76°C and 131.80°C, and the ignition temperatures were 337.74°C and 360.18°C, respectively. The indicator gas detection results showed that the first-appearance temperature of C_2_H_4_ was 85°C for the HQL and DY coals, whereas the first-appearance temperature of C_2_H_6_ varied: 115°C for the HQL coal and 130°C for the DY coal. The first-appearance temperatures of C_2_H_2_ were 180°C and 195°C for the HQL and DY coals, respectively. The experiments on coal oxidation spontaneous combustion showed that the spontaneous combustion period of the HQL and DY coals were 35.45 and 42.3 days, respectively. The heating process during combustion could be divided into four stages: a latent period of spontaneous combustion, a slow spontaneous heating period, an accelerated spontaneous heating period, and a period of combustion. The critical temperature of each stage showed a good correlation with the incipient temperature of the indicator gases, namely C_2_H_2_, C_2_H_4_, and C_2_H_6_, and the appearance of the above gases can be used to characterize the degree of spontaneous combustion of coal.

## Introduction

The spontaneous combustion of coal is a major disaster in the mining, storage, and transportation chain of coal production, restricting the development of the coal industry [1,2]. The spontaneous combustion of coal not only causes significant wastage of coal resources [3,4], but also leads to mine fires that vary the airflow in the mine ventilation system, resulting in gas explosions and casualties [5–7]. Moreover, SO_2_, NO_x_, tar, and other elements (such as As, Cd, Cr, Cu, and Pb) produced during the combustion pollute the atmosphere, water source, and soil, adversely affecting human health [8–10]. Therefore, it is significant to study the occurrence of the spontaneous combustion of coal and devise appropriate prevention measures.

The spontaneous combustion of coal is a process involving a complex chemical and physical reaction between coal and oxygen. This process is also affected by coal seam factors (coal rank, lithofacies, volatile substances, moisture, surface area, and sulfur content), geological factors (coal seam slope, coal seam thickness, cracks, and fissures), and mining factors (advancing speed, pillar and roof conditions, mining methods, ventilation, and old roadways) [11]. Its formation and development can be characterized by a spontaneous, slow, dynamically varying process involving exothermic reaction, heat build-up, and warming, causing combustion. The combustion process comprises a latent period [12], a self-heat period [13], and a combustion period [14]. Coal seams with a tendency to undergo spontaneous combustion require a certain amount of time after being mined and broken before spontaneously combusting [15–17]. Extensive studies have been conducted on the process of coal spontaneous combustion, its characteristic temperature, indices for combustion, division of the combustion stages, and early warning. Onifade et al. conducted a detailed evaluation of prediction techniques for spontaneous combustion commonly used in academia, research institutions, and industry [18]. Wen et al. built several large-scale experimental platforms of different scales to simulate the spontaneous combustion process of coal [19]. Deng et al. used a coal spontaneous combustion experimental setup to explore the effects of various factors on the minimum period of combustion [20,21]. The temperature of coal is the most direct and accurate indicator determining the degree of coal spontaneous combustion [22,23]. However, the coal wall and goaf of the coal are a relatively closed local space. It is almost impossible to accurately judge the combustion state of coal by directly measuring the temperature of the coal in these areas. There is a good correlation between the concentration of gas products and the coal temperature during the spontaneous combustion of coal [24–28], and the combustion stage can be predicted by monitoring the coal mine gases.

Despite the advancements made, research on the characteristic temperature of the spontaneous combustion of coal has mainly revolved around conducting natural ignition experiments and spontaneous combustion warming experiments, while various characteristic temperatures have been obtained by weight gain, weight loss, and weight loss rate using thermogravimetric analyses. Scholars demarcated 70°C as the self-heating temperature (SHT) of coal based on the temperature rise state of the spontaneous combustion process. The period in which the coal temperature is lower than the SHT is called the preparation period, and the period in which the coal temperature is between the SHT and the ignition temperature is called the self-heating period. The period in which the coal temperature reaches or exceeds the ignition temperature is called the combustion period. Through a thermogravimetric analysis, some scholars divided the characteristic temperature in the combustion process of coal samples into initial temperature, cracking temperature, growth temperature, ignition temperature, peak temperature, and burnout temperature, and divided the spontaneous combustion process into different stages [29,30]. The characteristic temperatures obtained from the different experiments are different, because of which the division of the characteristic temperature of spontaneous combustion becomes unclear. Moreover, studies on combustion indices have largely remained in the experimental stage given the difficulty in collecting and organizing on-site combustion data, and it remains difficult to apply experimental data to a coal mine site. In existing research, there are few reports on the division of the spontaneous combustion stage using the initial temperature of the index gas. Therefore, further studies are required on the division of the combustion process stages and the selection of combustion indices applicable to coal mine sites.

This study focused on the analysis of the characteristic temperature of coal spontaneous combustion, the generation law of the indicator gases generated during the spontaneous combustion, the combustion process, and the division of combustion stages. Nonstick coal and lignite coal samples were collected separately from the Hongqingliang mine in Ordos and the Dayan mine in Hulun Buir. Through field sample measurement, thermogravimetric analysis experiment, indicator gas detection experiment, and coal oxidative spontaneous combustion experiment, the different stages in the warming process of coal oxidative spontaneous combustion were explored. To divide each stage of the spontaneous combustion warming process, we established a method based on the relationship between the critical temperature of each stage of the warming process and the generation of indicator gases.

## Samples and methods

### Coal samples

Nonstick coal and lignite coal samples were collected separately from the Hongqingliang (HQL) mine in Ordos and the Dayan (DY) mine in Hulun Buir. Both the coal mines are located in Inner Mongolia Autonomous Region of China. The coal samples from the two mines were low metamorphic coal with a strong spontaneous combustion tendency, and the production process of the two mines has been threatened by coal spontaneous combustion to a certain extent. The originally collected coal samples were sealed and transported to a laboratory and processed in accordance with the preparation method of coal samples (GB474-2008). Coal samples with particle sizes ranging from 0.075 to 0.106 mm and 0.075 to 2 mm were selected for the thermogravimetric analysis experiments, indicator gas detection experiments, and coal oxidation spontaneous combustion experiment. Table 1 presents the specific information of the coal samples.

**Table 1:** Proximate analysis and metamorphic degree of the coal samples collected.

Coal sample	Industrial analysis/%				Rank
	*M*_*ad*_	*A*_*ad*_	*V*_*da*_	*FC*_*ad*_	
HQL	3.55	12.80	36.07	47.58	Nonstick coal
DY	9.12	8.01	37.88	44.99	Lignite

Note: HQL and DY represent the coal samples taken from the Hongqingliang and Dayan mines, respectively.

### Thermogravimetric analysis experiment

The STA449C synchronous thermal analyzer (made in Germany) was used to conduct thermogravimetric experiments on coal samples. Approximately 10 mg of the coal sample with a particle size range of 0.075–0.106 mm was taken each time for the test, and the coal samples were heated from 30°C to 800°C with a constant heating rate of 5°C/min and a volume ratio of 1:4 for the O_2_ to N_2_ mixture at a flow rate of 50 mL/min. The TG–DTG curves of the coal samples were obtained by recording the TG and DTG signals with the increase in the temperature of the coal samples. From the TG–DTG curve, the temperature describing the combustion process of the coal sample can be obtained, including the initial temperature denoted by *T*_*0*_, the pyrolysis temperature denoted by *T*_*1*_ (at the end of water evaporation and desorption, the coal starts to absorb oxygen and gain weight), the growth temperature denoted by *T*_*2*_ (temperature at the beginning of accelerated mass loss of the coal samples), the ignition temperature denoted by *T*_*ig*_, the peak temperature denoted by *T*_*max*_, and the temperature of burning out *T*_*b*_. The temperature corresponding to the maximum reaction rate point at the top of the DTG curve was *T*_*max*_. *T*_*ig*_ was the temperature at which coal started to burn, and this can be determined using the TG/DTG tangent method [29,30].

### Detection experiment on indicator gases

**Fig 1:**
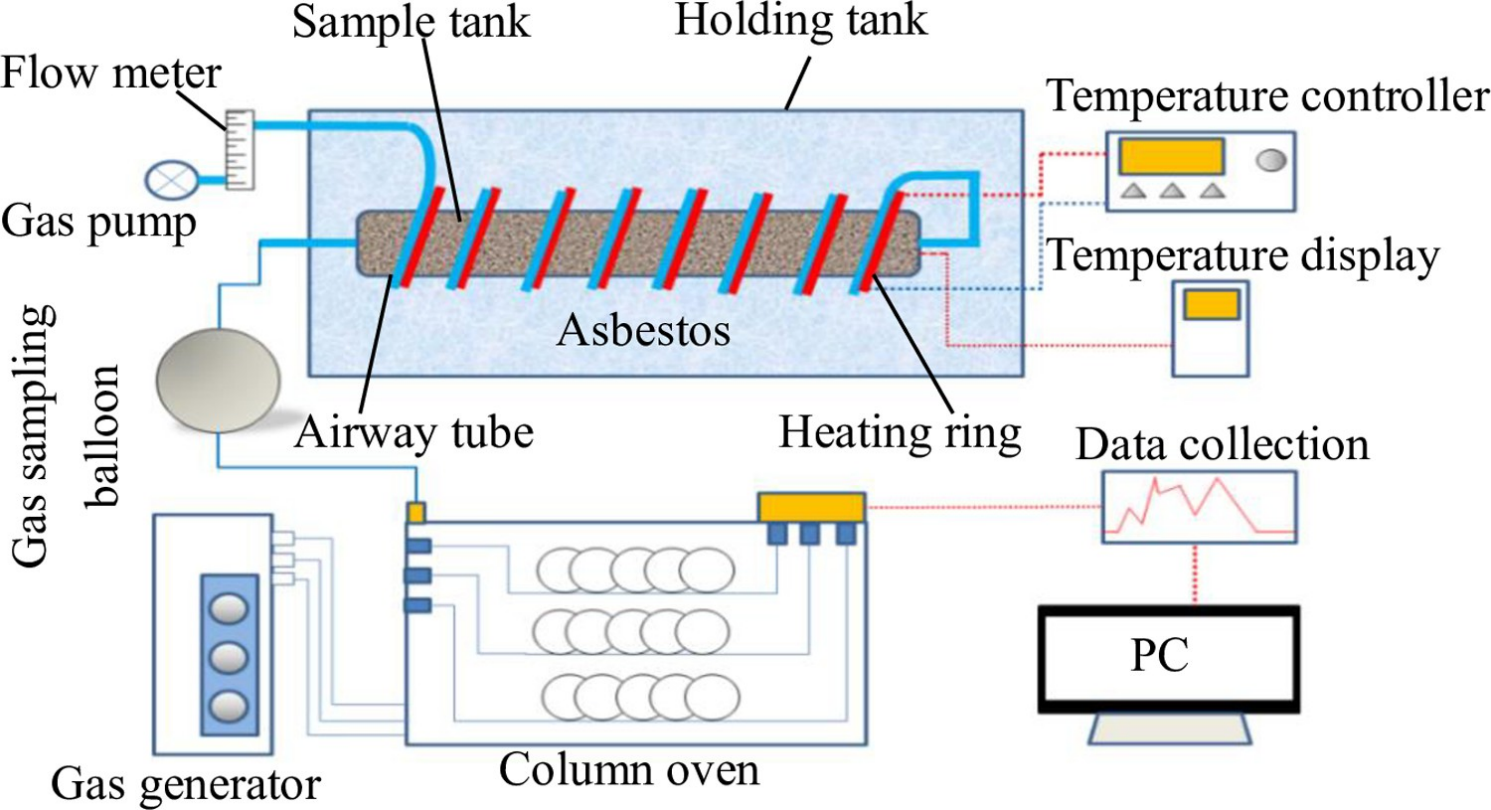
Schematic of the experimental setup for indicator gas detection.

Fig 1 shows the experimental device used for detecting the indicator gases. The experimental setup comprised a programmable temperature device and a chromatographic analysis device (KSS-5690 mining gas chromatograph). The programmable temperature device included a gas pump, a flow meter, a coal sample tank (inner diameter: 2.5 cm, length: 25 cm), a holding tank, a K-type thermocouple, a temperature measuring instrument, a temperature controller, a heating ring, and a gas-conducting steel pipe. The temperature controller was connected to the heating ring, and the heating power of the heating ring could be changed by adjusting the temperature controller to heat the gases in the gas-conducting steel pipe and the coal samples in the coal sample tank. The flow meter was set between the gas pump and the gas-conducting steel pipe, which was convenient for controlling the gas flow. The heating ring and the gas-conducting steel pipe were of a spiral structure, sleeved on the outer wall of the coal sample tank, and the gas outlet and gas inlet at both ends of the tank were connected using the gas-conducting steel pipe. The temperature measuring instrument was equipped with a K-type thermocouple to monitor the temperature of the coal in the sample tank. The sample tank was placed inside the holding tank, and the void was filled with asbestos for thermal insulation.

The main gas components produced in the process of coal oxidation and spontaneous combustion include CO, CO_2_, CH_4_, C_2_H_4_, C_2_H_6_, and C_2_H_2_. Among them, coal reacts with oxygen at ordinary temperatures to produce CO, CO_2_, and CH_4_ as well as including coal analysis. In the part of the index gas detection experiment, the aforementioned gases are not discussed. C_2_H_6_, C_2_H_4_, and C_2_H_2_ are important indicator gases of the spontaneous combustion of coal, and when the coal temperature rises to a certain level, these gases are produced due to pyrolysis reactions [31,32], and the gas concentration increases with the increase in the temperature. Therefore, the generation of C_2_H_6_, C_2_H_4_, and C_2_H_2_ can be used to characterize the degree of spontaneous combustion. The coal samples were detected for oxidized gases in the temperature range of 25–250°C to judge the initial temperatures of the three indicator gases, namely C_2_H_4_, C_2_H_6_, and C_2_H_2_. Fifty grams of the coal sample with a particle size range of 0.075–0.106 mm was placed in the coal sample tank. The air flow rate for the coal was set to 50 ml/min, and the sampling temperature interval was 5°C. The gases produced by combustion were collected in a gas pickup tank and detected by gas chromatography.

### Coal oxidation spontaneous combustion

**Fig 2:**
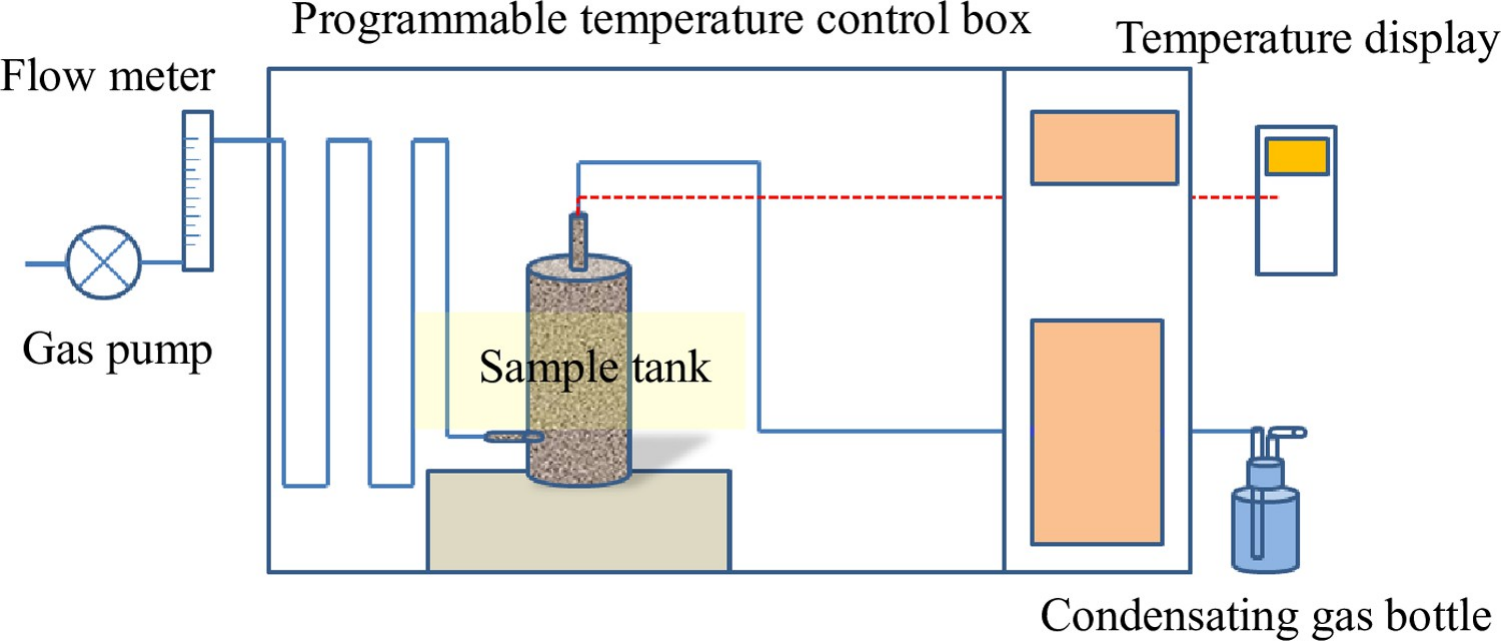
Experimental device for coal oxidation spontaneous combustion.

Fig 2 shows the experimental device for the coal oxidation spontaneous combustion, mainly comprising a gas supply pump, a flow meter, a programmable temperature control box, a sample tank, and a condensating gas bottle. All the parts were connected in series using a seamless steel pipe with an inner diameter of 4 mm, and each connection port was sealed with a high-temperature resistant sealant. A dry pipe was set in the gas supply pump to ensure the supply of dry gas in the experiment. To record the water production in the process of coal spontaneous combustion and oxidation, the exhaust pipeline of the coal sample tank was connected with the condensating gas bottle to remove the excess water in the gas. The K-type thermocouple temperature probe was buried at the center of the loose coal in the sample tank, and the temperature sensor was connected to the thermocouple to monitor the change in the coal temperature.

The experiment was conducted at a constant temperature of 16°C. The sample tank was a double-layer insulated steel bottle with a volume of 3.1 L. The mixed coal samples with a particle size range of 0.075–2 mm were placed in the tank, and the thermostat was started to adjust the temperature to preheat the coal samples to the temperature of the coal sample collection site. Subsequently, the gas supply pump was adjusted to pass in dry air at a flow rate of 40 ml/min. To prevent the temperature of the thermostat from exceeding the temperature of the coal samples, we took effective measures with heating the samples during the temperature rise of the coal spontaneous combustion experiments; the experimental process to maintain the thermostat temperature below the temperature of the coal sample should not be less than 2°C. The experiments were conducted with the environmental temperature of the coal sample collection site as the initial temperature, and the coal ignition temperature *T*_*ig*_ in the thermogravimetric experiment as the end temperature, during which the temperature data of the coal samples were recorded manually.

## Experimental results

### Analysis of characteristic temperature of coal combustion

**Fig 3:**
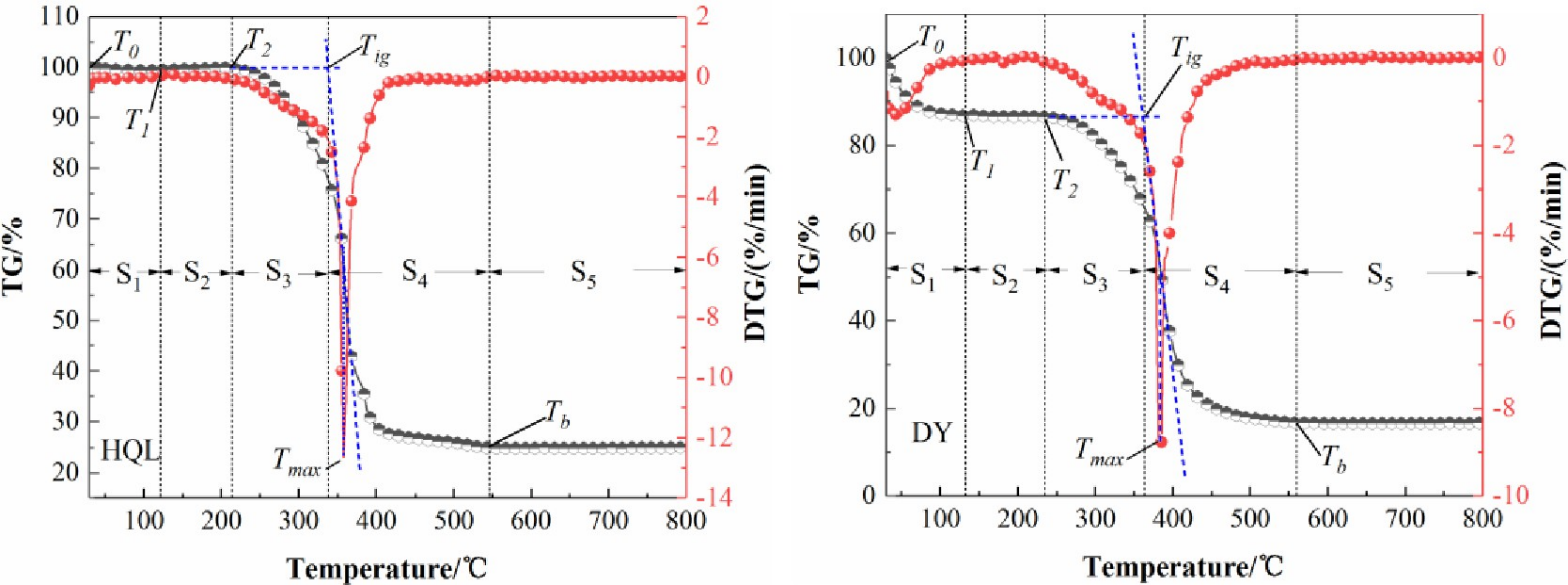
TG and DTG curves of the two coal samples studied.

Fig 3 shows the TG and DTG curves of the two coal samples studied. In the process of oxidative spontaneous combustion, different structures in the coal molecules are involved during the coal–oxygen reaction at a specific temperature [33]. Macroscopically, the samples exhibited a change in their weight loss rate and thermal weight loss. The corresponding temperature was called the characteristic temperature of the coal oxidation reaction process. Thus, the characteristic temperatures of the oxidation reaction of the two coal samples could be determined from the changes in the weight.

As shown in Fig 3, based on the weight gain and loss steps and the characteristic temperature of the coal samples in this study, the oxidation process can be divided into five stages [31]: the S_1_ water evaporation and gas desorption stage (*T*_*0*_−*T*_*1*_), S_2_ oxygen absorption and weight gain stage (*T*_*1*_−*T*_*2*_), S_3_ thermal decomposition and weight loss stage (*T*_*2*_−*T*_*ig*_), S_4_ combustion stage (*T*_*ig*_−*T*_*b*_), and S_5_ burnout stage (>*T*_*b*_). Table 2 presents the characteristic temperatures of the two coal samples studied. The cracking temperature *T*_*1*_ of the HQL coal sample was 115.76°C, and the ignition temperature *T*_*ig*_ was 337.74°C; those of the DY coal were 131.80°C and 360.18°C respectively, which can be obtained from the thermogravimetric experiments. Among them, the ignition temperature *T*_*ig*_ of the coal samples can be used as the final temperature of the coal oxidative spontaneous combustion experiment.

**Table 2:** Characteristic temperature of combustion of coal samples.

Sample	*T*_*0*_°C	*T*_*1*_°C	*T*_*2*_°C	*T*_*ig*_/°C	*T*_*max*_/°C	*T*_*b*_/°C
HQL	30.00	115.76	207.20	337.74	357.77	537.01
DY	30.00	131.80	230.37	360.18	385.41	554.45

Note: HQL and DY represent the coal samples taken from the Hongqingliang and Dayan mines, respectively.

### Relationships between the initial temperature of indicator gases and the temperature of coal

**Fig 4:**
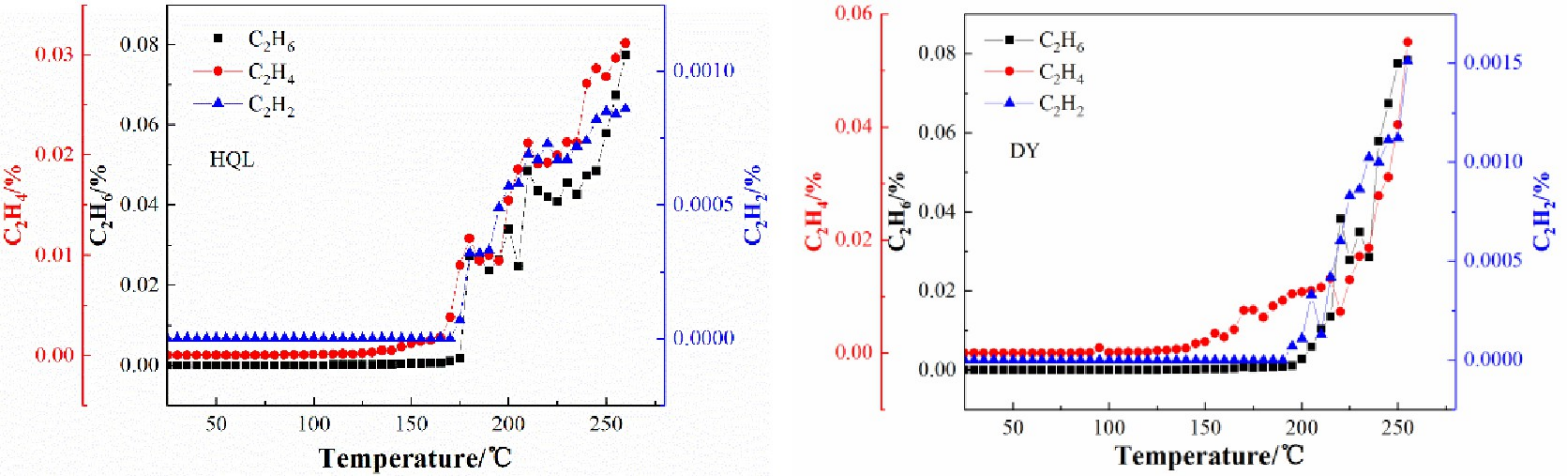
Variation in the concentration of representative gases with the oxidation temperature of the coal sample.

Fig 4 shows the relationships between the initial temperature of the indicator gases and the coal temperature. At the beginning of the experiment, the coal temperature was low, and the concentrations of C_2_H_4_, C_2_H_6_ and C_2_H_2_ were all 0. As the coal temperature increased, C_2_H_4_ was detected first, C_2_H_6_ was detected as the temperature increased further, and C_2_H_2_ was finally detected as the temperature continued to increase. The concentrations of the three indicator gases increased with the increase in the coal temperature. Table 3 presents the specific results of the first-appearance temperature and first-appearance concentration of the indicator gases during the warming of the coal samples. As listed in Table 3, the first-appearance temperature of C_2_H_4_ is 85°C for both the coal samples. The first-appearance temperatures of C_2_H_6_ are 115 and 130°C, respectively, and the first-appearance temperatures of C_2_H_2_ are respectively 180 and 195°C. Under the aforementioned conditions, the coal samples from HQL had slightly lower temperatures than those from DY.

**Table 3:** Indicative gas output results of coal samples in the heating stage.

Detected gases	HQL			DY		
	C_2_H_6_	C_2_H_4_	C_2_H_2_	C_2_H_6_	C_2_H_4_	C_2_H_2_
Initial temperature/°C	115	85	180	130	85	195
Initial concentration/%	0.00009	0.00004	0.00007	0.00006	0.00005	0.00007

Note: HQL and DY represent the coal samples taken from the Hongqingliang and Dayan mines, respectively.

There was an evident correlation between the initial temperatures of C_2_H_4_, C_2_H_6_, and C_2_H_2_ and the coal temperature. In the prevention and control of coal spontaneous combustion disasters, the temperature range of the high-temperature core of the coal can be determined by monitoring C_2_H_4_, C_2_H_6_, and C_2_H_2_.

### Stage division of coal spontaneous combustion heating process

**Fig 5:**
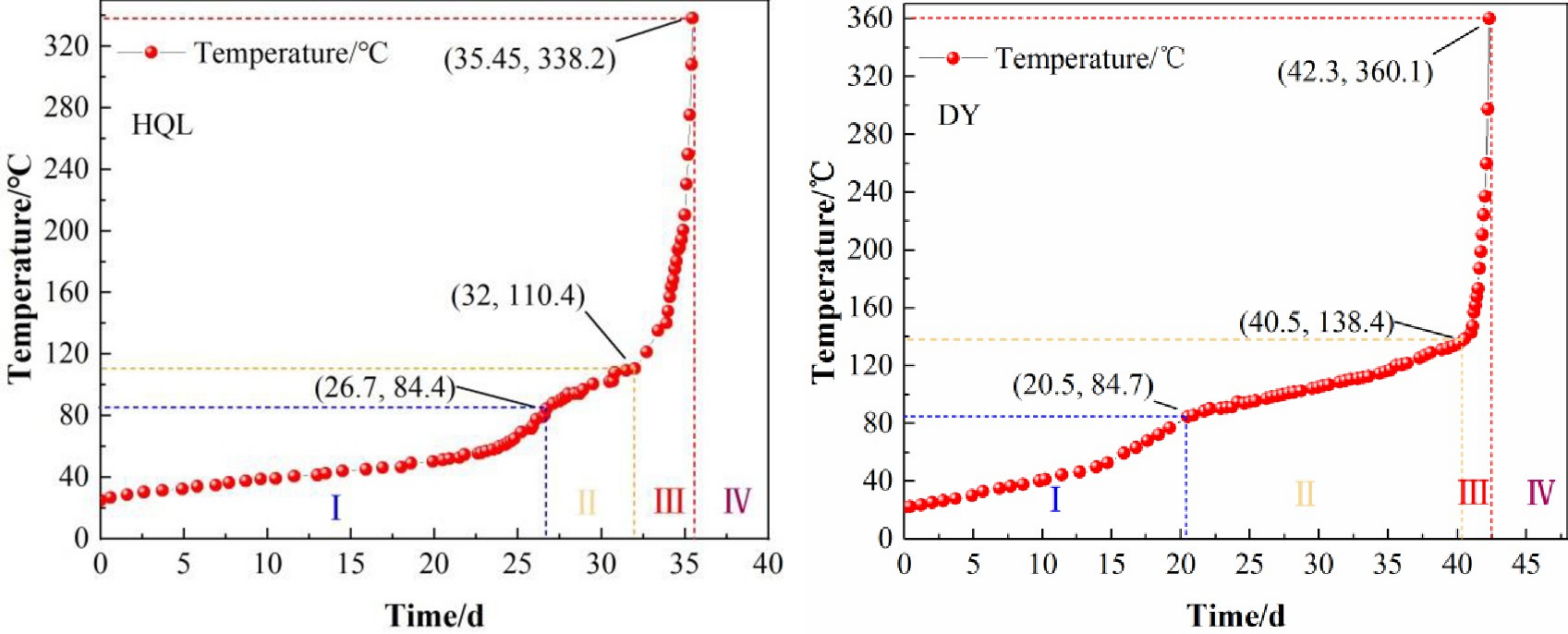
Upward trend of coal spontaneous combustion.

The starting temperature of the coal spontaneous combustion warming experiment was set as the temperature of the coal sample collection site, and the final temperature was the ignition temperature *T*_*ig*_ obtained from the thermogravimetric experiments. Fig 5 shows the variation in the coal temperature with time during the experiment.

The temperature rise of the coal spontaneous combustion experiments reflects the coal oxidation temperature rise process vividly. After analyzing the temperature data of the two coal samples, the following conclusions can be drawn: (1) The spontaneous combustion period of the HQL coal at temperatures ranging from 25°C to 338.2°C was approximately 35.45 days and that of the DY coal at temperatures ranging from 22°C to 360.1°C was approximately 42.3 days. (2) At the beginning of the coal oxidation spontaneous combustion experiments, the oxidation warming rate of the coal was low. When the temperature reached the first critical temperature of 85°C, the warming trend of the coal decreased significantly. (3) When the temperature reached the second critical temperature (110.4°C for the HQL coal and 138.4°C for the DY coal), the reaction between coal and oxygen was enhanced, and the warming trend of the coal increased significantly until the ignition temperature of the coal. Based on the changes in the warming trend, the coal oxidation spontaneous combustion process can be divided into four stages: I) latent period of spontaneous combustion, Ⅱe slow spontaneous heating period, Ⅲ) accelerated spontaneous heating period, and Ⅳe period of combustion. Table 4 presents the specific details of the four stages.

**Table 4:** Amount of condensed water precipitation during the upward trend of coal spontaneous combustion.

Stage	Description	HQL			DY		
		Temperature range/°C	Time/d	Condensate volume/ml	Temperature range/°C	Time/d	Condensate volume/ml
Ⅰ	Latent period of spontaneous combustion	25–84.4	26.7	14.5	22–84.7	20.5	10.6
Ⅱ	Slow spontaneous heating period	84.4–110.4	5.3	7.4	84.7–138.4	20.0	32.6
Ⅲ	Accelerated spontaneous heating period	110.4–338.2	3.45	1.8	138.4–360.1	1.8	1.2
Ⅳ	Period of combustion	>338.2	—	—	>360.1	—	—

Unlike the results of previous studies on the division of the combustion process stages, we divided the spontaneous heating period into two stages: a slow spontaneous heating period and an accelerated spontaneous heating period. It was presumed that this phenomenon was due to the evaporation of water in the coal to absorb heat. After the latent period of spontaneous ignition, the rate of coal oxidation reaction accelerated, the water generated by the decomposition of unstable oxides increased, and most of the heat released from the coal–reaction was used to evaporate the water remaining in the natural latent period of coal and the water generated by the coal oxidation reaction in the slow spontaneous heating period. This was macroscopically manifested in the reduced heating rate of the coal and the increase in the water precipitated from the coal, i.e., the slow self-heating stage. With the further development of the coal oxidation exothermic reaction, most of the water in the coal evaporated, and the accelerated spontaneous heating stage commenced, in which the coal oxidation exothermic intensity continued to increase, and most of the heat was accumulated in the coal except for the water produced by the evaporation reaction. This was macroscopically manifested in the accelerated heating of the coal, i.e., the accelerated spontaneous heating stage.

To verify the conjecture that water affects the division of the stages in the coal spontaneous heating period, the amount of condensed water in the condensate gas bottle during the combustion experiment was determined. Since the coal oxidative spontaneous combustion experiments were conducted at a constant temperature and the temperature was set to 16°C throughout, the condensate volume in the condensate gas bottle reflected the water production at each stage of the coal spontaneous combustion process. Table 4 presents the volume of the condensate collected during the warming phase of the spontaneous combustion for the two coal samples. To visually reflect the condensate production at each stage of the coal spontaneous combustion process, the average water production rate was introduced.


$$\bar{v}=\frac{Q}{\tau }$$ (1)

Here, $\bar{v}$ is the average value of the water collected in the condensate gas bottle with the unit of ml·d^−1^, *Q* is the total amount of water collected in the condensing gas bottle during the warming stage with the unit of ml, and *τ* is the time of the warming stage with the unit of d.

**Fig 6:**
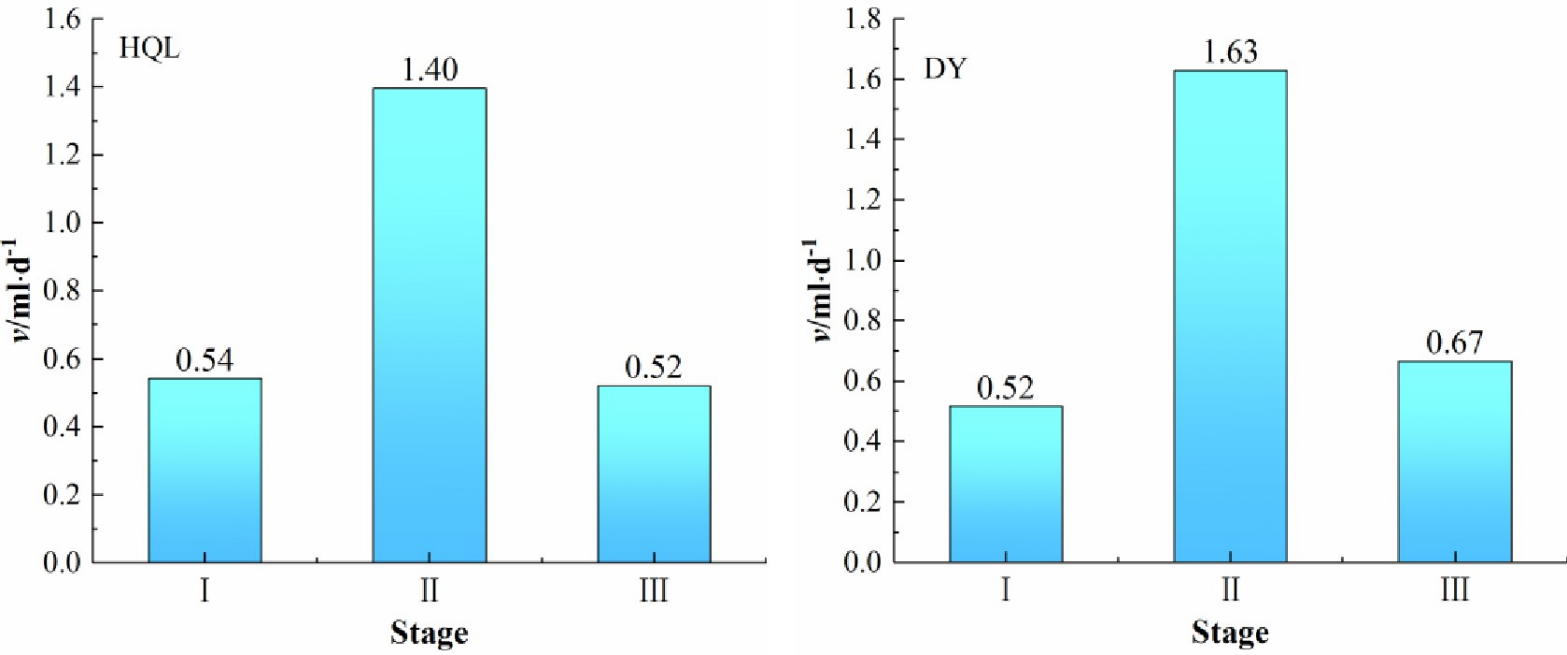
Comparison of the average water production rate in different stages of the upward process of coal spontaneous combustion.

Fig 6 shows a comparison of the average water production rate of the coal samples at each stage of the spontaneous combustion. The average rates of water production during the latent period of spontaneous combustion, slow spontaneous heating period, and accelerated spontaneous heating period were 0.54, 1.40 and 0.52 ml·d^−1^ for the HQL coal, respectively. The average rates for the DY coals were respectively 0.52, 1.63, and 0.67 ml·d^−1^. The rate of water production was maximum in the slow spontaneous heating period, indicating that most of the heat released from the coal–oxygen reaction was used to evaporate the water remaining in the latent period of the combustion and produced by the oxidation reaction of coal in the slow spontaneous heating period. Under the effect of heat absorption by water evaporation, the spontaneous heating period comprises two stages: a slow spontaneous heating period and an accelerated spontaneous heating period. The difference in the average rate of water production was also consistent with the field observation showing “first drops of water emergence, followed by the termination of water evaporation, and finally the commencement of either burning or smoking” in the coal spontaneous combustion.

## Division of coal spontaneous combustion stages and selection of indicator gases

**Fig 7:**
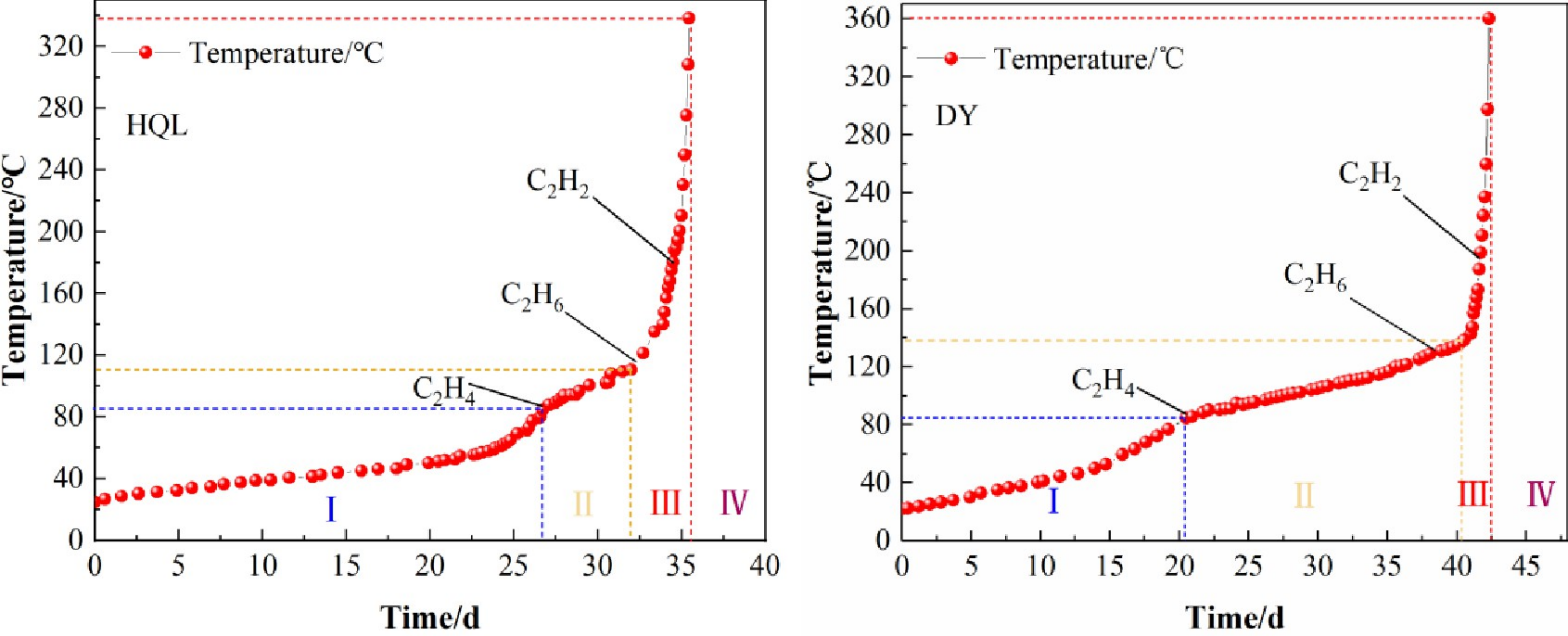
Upward trend of coal spontaneous combustion.

The results of the coal oxidation spontaneous combustion experiments, indicator gas detection experiments, and thermogravimetric analysis experiments were comprehensively analyzed. The critical temperature of the latent period of the spontaneous combustion and the slow spontaneous heating period of the HQL coal was 84.4°C and that of the DY coal was 84.7°C. The first-appearance temperature of C_2_H_4_ for the two coal samples was the same, 85°C. The critical temperature of the slow and accelerated spontaneous heating periods of the DY coal was 138.4°C, which was close to the initial temperature of C_2_H_6_ (130°C) and the cracking temperature of *T*_*1*_ (131.80°C). The critical temperature of both the HQL and DY coals occurred in the accelerated spontaneous heating period of the coal: 180 and 195°C, respectively. The results of the coal oxidation spontaneous combustion experiments showed that it took approximately 0.95 days for the temperature of the HQL coal to rise from 180°C to 338.2°C and approximately 0.6 days for the temperature of the DY coal to rise from 195 to 360.1°C. This indicates that the coal was already at the extreme spontaneous combustion risk when C_2_H_2_ was detected. Fig 7 shows the correspondence between the first-appearance temperatures of each indicator gas and the temperature rise process during the spontaneous combustion.

For the daily prevention and control of spontaneous combustion disasters in coal mines, CO, C_2_H_4_, C_2_H_6_, and C_2_H_2_ gases should be monitored in a focused manner. In the current coal mine safety regulations of China, it is clearly proposed that the maximum allowable concentration of CO in coal mines should not exceed 0.0024%. CO can be used as an indicator gas in the early stages of the spontaneous combustion process; necessary fire prevention measures should be taken when the detected concentration of CO is greater than 0.0024% to stop the further development of the combustion process. When C_2_H_4_ was detected, it meant that the high-temperature core of the coal would enter the spontaneous heating period, with the possibility of spontaneous combustion. When C_2_H_6_ was detected, the high-temperature core of the coal had reached the cracking temperature, and the coal was in the accelerated spontaneous heating stage. When C_2_H_2_ was detected, the high-temperature core of the coal body had entered the accelerated spontaneous heating period and was in the state of spontaneous combustion risk.

## Conclusions

Through on-site sample measurement, thermogravimetric analysis experiments, indicator gas detection experiments, and coal oxidative spontaneous combustion experiments, this study analyzed the characteristic temperatures of the spontaneous combustion, production laws of indicator gases, and period of oxidative spontaneous combustion of HQL and DY coal samples under experimental conditions. The relationship between the critical temperature and the production of indicator gases at each stage of the combustion process was determined. The conclusions drawn can be summarized as follows:

1. By analyzing the results of the coal oxidation spontaneous combustion experiments, the spontaneous combustion period of the HQL coal was found to be 35.45 days and that of the DY coal was 42.3 days. The heating process of the combustion could be divided into four stages: a latent period of spontaneous combustion, a slow spontaneous heating period, an accelerated spontaneous heating period, and a period of combustion. Under the effect of heat absorption by water evaporation, the spontaneous heating period of the coal showed two stages: a slow spontaneous heating period and an accelerated spontaneous heating period.
2. The production of C_2_H_2_, C_2_H_4_, and C_2_H_6_ correlated well with the temperature of the coal; this can be used to predict the temperature range of the high-temperature core during the spontaneous combustion.
3. Based on a comprehensive analysis of the relationship between the critical temperature of each stage of the spontaneous combustion and the generation of indicator gases, a method for dividing the stages of the combustion based on the indicator gases C_2_H_2_, C_2_H_4_, and C_2_H_6_ was proposed. This method can help grasp the spontaneous combustion state of the high-temperature core of coal, which can help predict the spontaneous combustion disaster and provide timely and reasonable prevention and control measures. The methods established in this study for the division of coal spontaneous combustion stages and the selection of indicator gases have certain reference for the prevention of coal spontaneous combustion disasters.

## Supporting information

S1 Table Data from the thermogravimetric analysis experiment. (DOCX)

S2 Table Indicative gas output results of coal samples in the heating stage. (DOCX)

S3 Table Temperature rising trend of coal spontaneous combustion. (DOCX)
