# Supplementary material for: Division of coal spontaneous combustion stages and selection of indicator gases
Source: PLoS One. 2022 Apr 27;17(4):e0267479. doi: 10.1371/journal.pone.0267479 (PMC9045653; doi:10.1371/journal.pone.0267479)
Supplement: S1 Table — (DOCX) [file pone.0267479.s001.docx]

**S1** **Table. Data from the thermogravimetric analysis experiment**

| **HQL** | | | **DYK** | | |
| --- | --- | --- | --- | --- | --- |
| **Temp/°C** | **Time/min** | **Mass/%** | **Temp/°C** | **Time/min** | **Mass/%** |
| 29.46 | 0 | 100.0878 | 29.46 | 0 | 100 |
| 30.58 | 0.5 | 99.9435 | 30.58 | 0.5 | 99.4517 |
| 31.53 | 1 | 99.9066 | 31.53 | 1 | 99.127 |
| 32.42 | 1.5 | 99.8831 | 32.42 | 1.5 | 98.6929 |
| 33.36 | 2 | 99.8587 | 33.36 | 2 | 98.145 |
| 34.42 | 2.5 | 99.8395 | 34.42 | 2.5 | 97.6067 |
| 35.56 | 3 | 99.808 | 35.62 | 3 | 96.984 |
| 37.51 | 3.5 | 99.8329 | 36.99 | 3.5 | 96.376 |
| 38.88 | 4 | 99.8195 | 38.6 | 4 | 95.756 |
| 40.01 | 4.5 | 99.8191 | 40.4 | 4.5 | 95.1051 |
| 41.48 | 5 | 99.7922 | 41.75 | 5 | 94.451 |
| 42.57 | 5.5 | 99.7906 | 42.4 | 5.5 | 93.8063 |
| 44.02 | 6 | 99.7838 | 44.62 | 6 | 93.141 |
| 45.74 | 6.5 | 99.7896 | 47.01 | 6.5 | 92.4813 |
| 47.73 | 7 | 99.7357 | 49.58 | 7 | 91.823 |
| 49.98 | 7.5 | 99.7302 | 52.31 | 7.5 | 91.2206 |
| 52.44 | 8 | 99.7081 | 55.15 | 8 | 90.676 |
| 55.06 | 8.5 | 99.6912 | 58.09 | 8.5 | 90.1859 |
| 57.85 | 9 | 99.6881 | 61.12 | 9 | 89.699 |
| 60.74 | 9.5 | 99.684 | 64.2 | 9.5 | 89.3123 |
| 63.76 | 10 | 99.6457 | 67.3 | 10 | 88.945 |
| 66.81 | 10.5 | 99.6041 | 70.44 | 10.5 | 88.6341 |
| 69.91 | 11 | 99.5928 | 73.57 | 11 | 88.359 |
| 73.01 | 11.5 | 99.552 | 76.68 | 11.5 | 88.1279 |
| 76.14 | 12 | 99.5328 | 79.78 | 12 | 87.951 |
| 79.27 | 12.5 | 99.523 | 82.88 | 12.5 | 87.808 |
| 82.4 | 13 | 99.5049 | 85.95 | 13 | 87.687 |
| 85.54 | 13.5 | 99.474 | 89.02 | 13.5 | 87.5296 |
| 88.65 | 14 | 99.4927 | 92.04 | 14 | 87.453 |
| 91.78 | 14.5 | 99.4442 | 95.05 | 14.5 | 87.367 |
| 94.86 | 15 | 99.414 | 98.03 | 15 | 87.266 |
| 97.94 | 15.5 | 99.4148 | 100.98 | 15.5 | 87.2285 |
| 100.97 | 16 | 99.4377 | 103.9 | 16 | 87.184 |
| 103.97 | 16.5 | 99.4192 | 106.79 | 16.5 | 87.125 |
| 106.96 | 17 | 99.4279 | 109.65 | 17 | 87.081 |
| 109.92 | 17.5 | 99.3991 | 112.47 | 17.5 | 87.0241 |
| 112.84 | 18 | 99.413 | 115.28 | 18 | 86.995 |
| 115.76 | 18.5 | 99.3883 | 118.07 | 18.5 | 86.9903 |
| 118.63 | 19 | 99.4044 | 120.84 | 19 | 86.922 |
| 121.49 | 19.5 | 99.4421 | 123.59 | 19.5 | 86.907 |
| 124.34 | 20 | 99.4764 | 126.33 | 20 | 86.856 |
| 127.16 | 20.5 | 99.5014 | 129.07 | 20.5 | 86.828 |
| 129.98 | 21 | 99.5114 | 131.8 | 21 | 86.851 |
| 132.79 | 21.5 | 99.5133 | 134.49 | 21.5 | 86.856 |
| 135.57 | 22 | 99.5464 | 137.2 | 22 | 86.859 |
| 138.36 | 22.5 | 99.5827 | 139.9 | 22.5 | 86.9034 |
| 141.11 | 23 | 99.6144 | 142.58 | 23 | 86.8223 |
| 143.86 | 23.5 | 99.6529 | 145.26 | 23.5 | 86.8648 |
| 146.59 | 24 | 99.6574 | 147.93 | 24 | 86.7565 |
| 149.32 | 24.5 | 99.6645 | 150.59 | 24.5 | 86.747 |
| 152.03 | 25 | 99.6764 | 153.23 | 25 | 86.764 |
| 154.73 | 25.5 | 99.6568 | 155.87 | 25.5 | 86.7279 |
| 157.44 | 26 | 99.6454 | 158.5 | 26 | 86.7143 |
| 160.12 | 26.5 | 99.6729 | 161.12 | 26.5 | 86.7036 |
| 162.79 | 27 | 99.7004 | 163.75 | 27 | 86.6198 |
| 165.46 | 27.5 | 99.7099 | 166.35 | 27.5 | 86.6515 |
| 168.1 | 28 | 99.7334 | 168.95 | 28 | 86.633 |
| 170.73 | 28.5 | 99.7295 | 171.56 | 28.5 | 86.671 |
| 173.37 | 29 | 99.7744 | 174.14 | 29 | 86.695 |
| 176 | 29.5 | 99.7768 | 176.73 | 29.5 | 86.7135 |
| 178.63 | 30 | 99.7864 | 179.32 | 30 | 86.638 |
| 181.26 | 30.5 | 99.7814 | 181.88 | 30.5 | 86.603 |
| 183.87 | 31 | 99.7974 | 184.47 | 31 | 86.5969 |
| 186.48 | 31.5 | 99.8459 | 187.04 | 31.5 | 86.6428 |
| 189.09 | 32 | 99.8734 | 189.61 | 32 | 86.6585 |
| 191.69 | 32.5 | 99.8814 | 192.18 | 32.5 | 86.6124 |
| 194.27 | 33 | 99.8814 | 194.75 | 33 | 86.6362 |
| 196.87 | 33.5 | 99.8834 | 197.31 | 33.5 | 86.635 |
| 199.46 | 34 | 99.8944 | 199.86 | 34 | 86.603 |
| 202.03 | 34.5 | 99.9034 | 202.42 | 34.5 | 86.5905 |
| 204.62 | 35 | 99.9254 | 204.98 | 35 | 86.620 |
| 207.2 | 35.5 | 99.8717 | 207.54 | 35.5 | 86.619 |
| 209.76 | 36 | 99.8534 | 210.07 | 36 | 86.603 |
| 212.32 | 36.5 | 99.8073 | 212.62 | 36.5 | 86.603 |
| 214.88 | 37 | 99.7754 | 215.16 | 37 | 86.603 |
| 217.44 | 37.5 | 99.7378 | 217.71 | 37.5 | 86.602 |
| 220 | 38 | 99.6684 | 220.24 | 38 | 86.612 |
| 222.55 | 38.5 | 99.6239 | 222.77 | 38.5 | 86.6109 |
| 225.11 | 39 | 99.5454 | 225.3 | 39 | 86.5933 |
| 227.66 | 39.5 | 99.4659 | 227.84 | 39.5 | 86.5985 |
| 230.19 | 40 | 99.3724 | 230.37 | 40 | 86.564 |
| 232.74 | 40.5 | 99.3 | 232.9 | 40.5 | 86.5134 |
| 235.29 | 41 | 99.1944 | 235.42 | 41 | 86.478 |
| 237.83 | 41.5 | 99.0594 | 237.94 | 41.5 | 86.4543 |
| 240.36 | 42 | 98.9234 | 240.47 | 42 | 86.397 |
| 242.89 | 42.5 | 98.7948 | 242.99 | 42.5 | 86.3481 |
| 245.43 | 43 | 98.6304 | 245.52 | 43 | 86.253 |
| 247.97 | 43.5 | 98.4408 | 248.04 | 43.5 | 86.2087 |
| 250.49 | 44 | 98.2714 | 250.56 | 44 | 86.137 |
| 253.03 | 44.5 | 98.053 | 253.08 | 44.5 | 86.0329 |
| 255.55 | 45 | 97.7914 | 255.6 | 45 | 85.899 |
| 258.08 | 45.5 | 97.5323 | 258.13 | 45.5 | 85.7921 |
| 260.62 | 46 | 97.2834 | 260.64 | 46 | 85.705 |
| 263.12 | 46.5 | 96.951 | 263.15 | 46.5 | 85.579 |
| 265.66 | 47 | 96.6474 | 265.67 | 47 | 85.428 |
| 268.18 | 47.5 | 96.2839 | 268.18 | 47.5 | 85.250 |
| 270.7 | 48 | 95.9044 | 270.69 | 48 | 85.040 |
| 273.22 | 48.5 | 95.5053 | 273.19 | 48.5 | 84.8231 |
| 275.74 | 49 | 95.0804 | 275.71 | 49 | 84.627 |
| 278.26 | 49.5 | 94.6041 | 278.2 | 49.5 | 84.3537 |
| 280.76 | 50 | 94.0944 | 280.72 | 50 | 84.095 |
| 283.25 | 50.5 | 93.6321 | 283.23 | 50.5 | 83.8084 |
| 285.76 | 51 | 93.1094 | 285.7 | 51 | 83.515 |
| 288.27 | 51.5 | 92.5435 | 288.2 | 51.5 | 83.1947 |
| 290.75 | 52 | 91.9844 | 290.7 | 52 | 82.849 |
| 293.24 | 52.5 | 91.4797 | 293.19 | 52.5 | 82.4357 |
| 295.72 | 53 | 90.8744 | 295.68 | 53 | 82.051 |
| 298.2 | 53.5 | 90.2803 | 298.17 | 53.5 | 81.7074 |
| 300.69 | 54 | 89.5934 | 300.64 | 54 | 81.297 |
| 303.16 | 54.5 | 89.0328 | 303.12 | 54.5 | 80.8485 |
| 305.64 | 55 | 88.3434 | 305.58 | 55 | 80.328 |
| 308.1 | 55.5 | 87.7523 | 308.05 | 55.5 | 79.874 |
| 310.57 | 56 | 86.9904 | 310.54 | 56 | 79.427 |
| 313.03 | 56.5 | 86.3087 | 313 | 56.5 | 78.9196 |
| 315.51 | 57 | 85.6594 | 315.46 | 57 | 78.401 |
| 317.96 | 57.5 | 84.9191 | 317.93 | 57.5 | 77.8919 |
| 320.44 | 58 | 84.1584 | 320.4 | 58 | 77.337 |
| 322.91 | 58.5 | 83.4072 | 322.87 | 58.5 | 76.8118 |
| 325.38 | 59 | 82.6094 | 325.33 | 59 | 76.185 |
| 327.84 | 59.5 | 81.7629 | 327.81 | 59.5 | 75.6526 |
| 330.3 | 60 | 80.8974 | 330.27 | 60 | 75.087 |
| 332.77 | 60.5 | 79.9587 | 332.74 | 60.5 | 74.4623 |
| 335.25 | 61 | 79.0014 | 335.21 | 61 | 73.848 |
| 337.74 | 61.5 | 77.9995 | 337.7 | 61.5 | 73.2052 |
| 340.22 | 62 | 76.9314 | 340.18 | 62 | 72.5602 |
| 342.71 | 62.5 | 75.7509 | 342.67 | 62.5 | 71.8885 |
| 345.2 | 63 | 74.4244 | 345.17 | 63 | 71.139 |
| 347.69 | 63.5 | 72.9878 | 347.66 | 63.5 | 70.3929 |
| 350.2 | 64 | 71.2894 | 350.14 | 64 | 69.644 |
| 352.7 | 64.5 | 69.3133 | 352.65 | 64.5 | 68.7838 |
| 355.23 | 65 | 66.2864 | 355.16 | 65 | 67.968 |
| 357.77 | 65.5 | 59.5394 | 357.67 | 65.5 | 67.0618 |
| 360.3 | 66 | 53.6574 | 360.18 | 66 | 66.124 |
| 362.87 | 66.5 | 48.6666 | 362.71 | 66.5 | 65.0991 |
| 365.47 | 67 | 45.2354 | 365.23 | 67 | 64.016 |
| 368.09 | 67.5 | 42.9576 | 367.77 | 67.5 | 62.8045 |
| 370.8 | 68 | 41.0944 | 370.33 | 68 | 61.421 |
| 373.86 | 68.5 | 39.6131 | 372.9 | 68.5 | 59.8042 |
| 378.48 | 69 | 38.0374 | 375.5 | 69 | 58.035 |
| 382.05 | 69.5 | 36.7683 | 378.15 | 69.5 | 55.3249 |
| 384.35 | 70 | 35.5134 | 381.04 | 70 | 49.323 |
| 385.6 | 70.5 | 34.4107 | 385.44 | 70.5 | 46.5619 |
| 386.63 | 71 | 33.3654 | 387.49 | 71 | 44.222 |
| 388.1 | 71.5 | 32.4343 | 389.04 | 71.5 | 41.9761 |
| 390.02 | 72 | 31.6114 | 391.05 | 72 | 39.687 |
| 392.18 | 72.5 | 30.8822 | 393.33 | 72.5 | 37.6453 |
| 394.43 | 73 | 30.2344 | 395.66 | 73 | 35.688 |
| 396.75 | 73.5 | 29.6993 | 397.94 | 73.5 | 33.9562 |
| 399.11 | 74 | 29.2694 | 400.2 | 74 | 32.397 |
| 401.5 | 74.5 | 28.8525 | 402.47 | 74.5 | 31.0663 |
| 403.92 | 75 | 28.5384 | 404.77 | 75 | 29.853 |
| 406.35 | 75.5 | 28.2484 | 407.1 | 75.5 | 28.6868 |
| 408.79 | 76 | 28.0104 | 409.44 | 76 | 27.687 |
| 411.23 | 76.5 | 27.8473 | 411.81 | 76.5 | 26.8384 |
| 413.67 | 77 | 27.7214 | 414.19 | 77 | 26.043 |
| 416.14 | 77.5 | 27.6134 | 416.62 | 77.5 | 25.3173 |
| 418.62 | 78 | 27.5184 | 419.06 | 78 | 24.682 |
| 421.09 | 78.5 | 27.4362 | 421.5 | 78.5 | 24.1341 |
| 423.59 | 79 | 27.3844 | 423.98 | 79 | 23.573 |
| 426.1 | 79.5 | 27.3429 | 426.45 | 79.5 | 23.0684 |
| 428.61 | 80 | 27.2934 | 428.94 | 80 | 22.673 |
| 431.12 | 80.5 | 27.2043 | 431.44 | 80.5 | 22.3002 |
| 433.65 | 81 | 27.1634 | 433.93 | 81 | 21.941 |
| 436.18 | 81.5 | 27.0783 | 436.43 | 81.5 | 21.6026 |
| 438.73 | 82 | 27.0144 | 438.95 | 82 | 21.317 |
| 441.28 | 82.5 | 26.9623 | 441.47 | 82.5 | 21.067 |
| 443.82 | 83 | 26.9214 | 443.96 | 83 | 20.807 |
| 446.36 | 83.5 | 26.8342 | 446.48 | 83.5 | 20.5695 |
| 448.9 | 84 | 26.8194 | 449 | 84 | 20.334 |
| 451.42 | 84.5 | 26.7219 | 451.52 | 84.5 | 20.1337 |
| 453.97 | 85 | 26.7144 | 454.04 | 85 | 19.932 |
| 456.51 | 85.5 | 26.6849 | 456.55 | 85.5 | 19.7469 |
| 459.04 | 86 | 26.6114 | 459.07 | 86 | 19.561 |
| 461.57 | 86.5 | 26.5235 | 461.62 | 86.5 | 19.4002 |
| 464.1 | 87 | 26.4984 | 464.12 | 87 | 19.257 |
| 466.62 | 87.5 | 26.4519 | 466.64 | 87.5 | 19.0907 |
| 469.16 | 88 | 26.3984 | 469.16 | 88 | 18.948 |
| 471.67 | 88.5 | 26.3674 | 471.67 | 88.5 | 18.7935 |
| 474.2 | 89 | 26.3194 | 474.2 | 89 | 18.688 |
| 476.73 | 89.5 | 26.278 | 476.7 | 89.5 | 18.5259 |
| 479.25 | 90 | 26.2144 | 479.23 | 90 | 18.449 |
| 481.78 | 90.5 | 26.2264 | 481.74 | 90.5 | 18.3445 |
| 484.29 | 91 | 26.1614 | 484.25 | 91 | 18.267 |
| 486.8 | 91.5 | 26.1334 | 486.75 | 91.5 | 18.1534 |
| 489.32 | 92 | 26.1014 | 489.26 | 92 | 18.061 |
| 491.83 | 92.5 | 26.0527 | 491.78 | 92.5 | 18.0034 |
| 494.36 | 93 | 26.0294 | 494.29 | 93 | 17.925 |
| 496.87 | 93.5 | 26.0164 | 496.8 | 93.5 | 17.9115 |
| 499.38 | 94 | 25.9984 | 499.31 | 94 | 17.823 |
| 501.9 | 94.5 | 25.9208 | 501.83 | 94.5 | 17.7605 |
| 504.4 | 95 | 25.8494 | 504.34 | 95 | 17.706 |
| 506.91 | 95.5 | 25.8019 | 506.85 | 95.5 | 17.6803 |
| 509.43 | 96 | 25.8014 | 509.36 | 96 | 17.6401 |
| 511.94 | 96.5 | 25.6638 | 511.87 | 96.5 | 17.568 |
| 514.46 | 97 | 25.6114 | 514.39 | 97 | 17.523 |
| 516.97 | 97.5 | 25.5683 | 516.9 | 97.5 | 17.438 |
| 519.48 | 98 | 25.4874 | 519.41 | 98 | 17.41 |
| 521.98 | 98.5 | 25.3737 | 521.91 | 98.5 | 17.3575 |
| 524.48 | 99 | 25.3484 | 524.43 | 99 | 17.327 |
| 527 | 99.5 | 25.2654 | 526.93 | 99.5 | 17.257 |
| 529.5 | 100 | 25.2054 | 529.45 | 100 | 17.202 |
| 532 | 100.5 | 25.1673 | 531.95 | 100.5 | 17.159 |
| 534.5 | 101 | 25.1034 | 534.45 | 101 | 17.109 |
| 537.01 | 101.5 | 25.0628 | 536.96 | 101.5 | 17.0316 |
| 539.51 | 102 | 25.0224 | 539.46 | 102 | 16.977 |
| 542.02 | 102.5 | 25.0036 | 541.95 | 102.5 | 16.983 |
| 544.52 | 103 | 25.0034 | 544.46 | 103 | 16.91 |
| 547 | 103.5 | 24.9644 | 546.96 | 103.5 | 16.887 |
| 549.51 | 104 | 24.9354 | 549.45 | 104 | 16.871 |
| 552 | 104.5 | 24.8899 | 551.96 | 104.5 | 16.8189 |
| 554.5 | 105 | 24.9094 | 554.45 | 105 | 16.814 |
| 556.97 | 105.5 | 24.9303 | 556.95 | 105.5 | 16.7706 |
| 559.48 | 106 | 24.9344 | 559.46 | 106 | 16.795 |
| 562 | 106.5 | 24.9344 | 561.96 | 106.5 | 16.7785 |
| 564.5 | 107 | 24.9224 | 564.47 | 107 | 16.748 |
| 567.01 | 107.5 | 24.9028 | 566.97 | 107.5 | 16.728 |
| 569.51 | 108 | 24.9184 | 569.48 | 108 | 16.74 |
| 572.02 | 108.5 | 24.9113 | 571.98 | 108.5 | 16.7239 |
| 574.52 | 109 | 24.9424 | 574.5 | 109 | 16.703 |
| 577.02 | 109.5 | 24.8782 | 577 | 109.5 | 16.703 |
| 579.53 | 110 | 24.8674 | 579.51 | 110 | 16.731 |
| 582.03 | 110.5 | 24.9243 | 582.01 | 110.5 | 16.6915 |
| 584.55 | 111 | 24.9244 | 584.54 | 111 | 16.685 |
| 587.03 | 111.5 | 24.9059 | 587.04 | 111.5 | 16.718 |
| 589.55 | 112 | 24.9244 | 589.54 | 112 | 16.694 |
| 592.05 | 112.5 | 24.9089 | 592.05 | 112.5 | 16.6674 |
| 594.57 | 113 | 24.9194 | 594.56 | 113 | 16.658 |
| 597.07 | 113.5 | 24.8981 | 597.07 | 113.5 | 16.6779 |
| 599.57 | 114 | 24.9094 | 599.58 | 114 | 16.656 |
| 602.07 | 114.5 | 24.9033 | 602.08 | 114.5 | 16.6688 |
| 604.59 | 115 | 24.8884 | 604.59 | 115 | 16.648 |
| 607.09 | 115.5 | 24.9144 | 607.1 | 115.5 | 16.65 |
| 609.59 | 116 | 24.9144 | 609.6 | 116 | 16.653 |
| 612.09 | 116.5 | 24.9479 | 612.09 | 116.5 | 16.6518 |
| 614.61 | 117 | 24.9474 | 614.61 | 117 | 16.666 |
| 617.12 | 117.5 | 24.9444 | 617.11 | 117.5 | 16.6501 |
| 619.63 | 118 | 24.9314 | 619.63 | 118 | 16.648 |
| 622.14 | 118.5 | 24.9122 | 622.14 | 118.5 | 16.6719 |
| 624.64 | 119 | 24.9134 | 624.64 | 119 | 16.615 |
| 627.15 | 119.5 | 24.9033 | 627.16 | 119.5 | 16.7015 |
| 629.66 | 120 | 24.8854 | 629.65 | 120 | 16.682 |
| 632.15 | 120.5 | 24.9352 | 632.17 | 120.5 | 16.7025 |
| 634.68 | 121 | 24.8964 | 634.67 | 121 | 16.708 |
| 637.18 | 121.5 | 24.8642 | 637.18 | 121.5 | 16.679 |
| 639.69 | 122 | 24.8914 | 639.7 | 122 | 16.697 |
| 642.19 | 122.5 | 24.8804 | 642.19 | 122.5 | 16.6648 |
| 644.7 | 123 | 24.8934 | 644.7 | 123 | 16.675 |
| 647.21 | 123.5 | 24.8644 | 647.21 | 123.5 | 16.662 |
| 649.71 | 124 | 24.8634 | 649.72 | 124 | 16.63 |
| 652.22 | 124.5 | 24.888 | 652.22 | 124.5 | 16.6279 |
| 654.72 | 125 | 24.8714 | 654.71 | 125 | 16.675 |
| 657.21 | 125.5 | 24.8644 | 657.22 | 125.5 | 16.6709 |
| 659.73 | 126 | 24.8644 | 659.72 | 126 | 16.672 |
| 662.23 | 126.5 | 24.8754 | 662.23 | 126.5 | 16.6573 |
| 664.73 | 127 | 24.8814 | 664.74 | 127 | 16.638 |
| 667.24 | 127.5 | 24.8825 | 667.25 | 127.5 | 16.638 |
| 669.75 | 128 | 24.8484 | 669.75 | 128 | 16.638 |
| 672.25 | 128.5 | 24.8495 | 672.26 | 128.5 | 16.638 |
| 674.75 | 129 | 24.8974 | 674.76 | 129 | 16.638 |
| 677.26 | 129.5 | 24.8648 | 677.26 | 129.5 | 16.629 |
| 679.76 | 130 | 24.8504 | 679.76 | 130 | 16.625 |
| 682.26 | 130.5 | 24.8683 | 682.26 | 130.5 | 16.6412 |
| 684.76 | 131 | 24.8804 | 684.77 | 131 | 16.653 |
| 687.28 | 131.5 | 24.8975 | 687.28 | 131.5 | 16.642 |
| 689.78 | 132 | 24.9044 | 689.8 | 132 | 16.638 |
| 692.28 | 132.5 | 24.9105 | 692.3 | 132.5 | 16.6471 |
| 694.79 | 133 | 24.9104 | 694.81 | 133 | 16.638 |
| 697.29 | 133.5 | 24.9198 | 697.31 | 133.5 | 16.6508 |
| 699.79 | 134 | 24.9164 | 699.81 | 134 | 16.674 |
| 702.3 | 134.5 | 24.9104 | 702.33 | 134.5 | 16.6761 |
| 704.8 | 135 | 24.9254 | 704.83 | 135 | 16.697 |
| 707.31 | 135.5 | 24.9372 | 707.34 | 135.5 | 16.667 |
| 709.82 | 136 | 24.9014 | 709.84 | 136 | 16.664 |
| 712.33 | 136.5 | 24.9185 | 712.36 | 136.5 | 16.656 |
| 714.82 | 137 | 24.8994 | 714.84 | 137 | 16.627 |
| 717.35 | 137.5 | 24.9094 | 717.36 | 137.5 | 16.624 |
| 719.85 | 138 | 24.9344 | 719.87 | 138 | 16.654 |
| 722.35 | 138.5 | 24.9144 | 722.36 | 138.5 | 16.666 |
| 724.86 | 139 | 24.9354 | 724.87 | 139 | 16.643 |
| 727.37 | 139.5 | 24.9614 | 727.37 | 139.5 | 16.6589 |
| 729.87 | 140 | 24.9664 | 729.88 | 140 | 16.652 |
| 732.37 | 140.5 | 24.9722 | 732.39 | 140.5 | 16.643 |
| 734.88 | 141 | 24.9664 | 734.89 | 141 | 16.658 |
| 737.39 | 141.5 | 24.9594 | 737.4 | 141.5 | 16.6265 |
| 739.9 | 142 | 24.9594 | 739.91 | 142 | 16.671 |
| 742.4 | 142.5 | 24.9603 | 742.41 | 142.5 | 16.6709 |
| 744.91 | 143 | 24.9614 | 744.92 | 143 | 16.658 |
| 747.41 | 143.5 | 24.9604 | 747.42 | 143.5 | 16.6471 |
| 749.91 | 144 | 24.9754 | 749.93 | 144 | 16.678 |
| 752.42 | 144.5 | 24.9889 | 752.42 | 144.5 | 16.678 |
| 754.92 | 145 | 25.0014 | 754.94 | 145 | 16.728 |
| 757.43 | 145.5 | 24.9995 | 757.43 | 145.5 | 16.6739 |
| 759.94 | 146 | 25.0124 | 759.95 | 146 | 16.677 |
| 762.44 | 146.5 | 24.9859 | 762.45 | 146.5 | 16.686 |
| 764.95 | 147 | 24.9794 | 764.95 | 147 | 16.706 |
| 767.45 | 147.5 | 24.9811 | 767.46 | 147.5 | 16.7095 |
| 769.95 | 148 | 25.0164 | 769.95 | 148 | 16.718 |
| 772.47 | 148.5 | 24.9894 | 772.46 | 148.5 | 16.72 |
| 774.97 | 149 | 24.9894 | 774.97 | 149 | 16.726 |
| 777.47 | 149.5 | 24.9894 | 777.48 | 149.5 | 16.7154 |
| 779.98 | 150 | 24.9995 | 779.98 | 150 | 16.709 |
| 782.47 | 150.5 | 25.0124 | 782.48 | 150.5 | 16.7062 |
| 784.98 | 151 | 24.9859 | 785 | 151 | 16.733 |
| 787.48 | 151.5 | 24.9794 | 787.51 | 151.5 | 16.6876 |
| 789.99 | 152 | 24.9811 | 790.01 | 152 | 16.711 |
| 792.5 | 152.5 | 25.0164 | 792.52 | 152.5 | 16.718 |
| 795 | 153 | 24.9894 | 795.01 | 153 | 16.711 |
