# Supplementary material for: Division of coal spontaneous combustion stages and selection of indicator gases
Source: PLoS One. 2022 Apr 27;17(4):e0267479. doi: 10.1371/journal.pone.0267479 (PMC9045653; doi:10.1371/journal.pone.0267479)
Supplement: S2 Table — (DOCX) [file pone.0267479.s002.docx]

**S2** **Table. Indicative gas output results of coal samples in the heating stage**

| **HQL** | | | | **DYK** | | | |
| --- | --- | --- | --- | --- | --- | --- | --- |
| **Temp/°C** | **C_2_H_6_/%** | **C_2_H_4_/%** | **C_2_H_2_/%** | **Temp/°C** | **C_2_H_6_/%** | **C_2_H_4_/%** | **C_2_H_2_/%** |
| 25 | 0 | 0 | 0 | 25 | 0 | 0 | 0 |
| 30 | 0 | 0 | 0 | 30 | 0 | 0 | 0 |
| 35 | 0 | 0 | 0 | 35 | 0 | 0 | 0 |
| 40 | 0 | 0 | 0 | 40 | 0 | 0 | 0 |
| 45 | 0 | 0 | 0 | 45 | 0 | 0 | 0 |
| 50 | 0 | 0 | 0 | 50 | 0 | 0 | 0 |
| 55 | 0 | 0 | 0 | 55 | 0 | 0 | 0 |
| 60 | 0 | 0 | 0 | 60 | 0 | 0 | 0 |
| 65 | 0 | 0 | 0 | 65 | 0 | 0 | 0 |
| 70 | 0 | 0 | 0 | 70 | 0 | 0 | 0 |
| 75 | 0 | 0 | 0 | 75 | 0 | 0 | 0 |
| 80 | 0 | 0 | 0 | 80 | 0 | 0 | 0 |
| 85 | 0 | 0.00004 | 0 | 85 | 0 | 0.00005 | 0 |
| 90 | 0 | 0.00005 | 0 | 90 | 0 | 0.00007 | 0 |
| 95 | 0 | 0.00006 | 0 | 95 | 0 | 0.0009 | 0 |
| 100 | 0 | 0.00008 | 0 | 100 | 0 | 0.00012 | 0 |
| 105 | 0 | 0.0001 | 0 | 105 | 0 | 0.00014 | 0 |
| 110 | 0 | 0.00012 | 0 | 110 | 0 | 0.00016 | 0 |
| 115 | 0.00009 | 0.00015 | 0 | 115 | 0 | 0.00018 | 0 |
| 120 | 0.00012 | 0.00012 | 0 | 120 | 0 | 0.0002 | 0 |
| 125 | 0.00013 | 0.00018 | 0 | 125 | 0 | 0.00046 | 0 |
| 130 | 0.00015 | 0.00028 | 0 | 130 | 0.00006 | 0.00054 | 0 |
| 135 | 0.00024 | 0.00048 | 0 | 135 | 0.0001 | 0.00068 | 0 |
| 140 | 0.00023 | 0.00049 | 0 | 140 | 0.00013 | 0.00081 | 0 |
| 145 | 0.00038 | 0.00086 | 0 | 145 | 0.00019 | 0.00168 | 0 |
| 150 | 0.00045 | 0.00118 | 0 | 150 | 0.00023 | 0.00201 | 0 |
| 155 | 0.00053 | 0.00135 | 0 | 155 | 0.00027 | 0.00351 | 0 |
| 160 | 0.00057 | 0.00151 | 0 | 160 | 0.00024 | 0.00283 | 0 |
| 165 | 0.00058 | 0.00178 | 0 | 165 | 0.00034 | 0.00416 | 0 |
| 170 | 0.00107 | 0.00381 | 0 | 170 | 0.00075 | 0.00753 | 0 |
| 175 | 0.00173 | 0.00901 | 0 | 175 | 0.00051 | 0.00764 | 0 |
| 180 | 0.02713 | 0.02165 | 0.00007 | 180 | 0.00054 | 0.00627 | 0 |
| 185 | 0.02642 | 0.00945 | 0.00032 | 185 | 0.00069 | 0.00831 | 0 |
| 190 | 0.02369 | 0.00995 | 0.00032 | 190 | 0.00083 | 0.00932 | 0 |
| 195 | 0.02642 | 0.00945 | 0.00013 | 195 | 0.0011 | 0.01045 | 0.00007 |
| 200 | 0.03401 | 0.01547 | 0.00049 | 200 | 0.00274 | 0.01082 | 0.00011 |
| 205 | 0.02467 | 0.01085 | 0.00097 | 205 | 0.00589 | 0.01102 | 0.00033 |
| 210 | 0.04851 | 0.02115 | 0.00048 | 210 | 0.0104 | 0.01163 | 0.00013 |
| 215 | 0.04348 | 0.01909 | 0.00099 | 215 | 0.01346 | 0.01319 | 0.00042 |
| 220 | 0.02053 | 0.01218 | 0.00077 | 220 | 0.03816 | 0.00733 | 0.0006 |
| 225 | 0.00875 | 0.00998 | 0.00053 | 225 | 0.02784 | 0.01298 | 0.00083 |
| 230 | 0.0146 | 0.0128 | 0.00057 | 230 | 0.03484 | 0.01713 | 0.00086 |
| 235 | 0.01161 | 0.01236 | 0.00047 | 235 | 0.02852 | 0.0186 | 0.00102 |
| 240 | 0.0228 | 0.01713 | 0.00052 | 240 | 0.05784 | 0.02779 | 0.001 |
| 245 | 0.02852 | 0.0186 | 0.00054 | 245 | 0.06734 | 0.03115 | 0.00111 |
| 250 | 0.05784 | 0.02779 | 0.00042 | 250 | 0.07744 | 0.04045 | 0.00112 |
