# Supplementary material for: Division of coal spontaneous combustion stages and selection of indicator gases
Source: PLoS One. 2022 Apr 27;17(4):e0267479. doi: 10.1371/journal.pone.0267479 (PMC9045653; doi:10.1371/journal.pone.0267479)
Supplement: S3 Table — (DOCX) [file pone.0267479.s003.docx]

**S3 Table.Temperature rising trend of coal spontaneous combustion**

| **HQL** |  | **DYK** |  |
| --- | --- | --- | --- |
| **Time/day** | **Temp/°C** | **Time/day** | **Temp/°C** |
| 0 | 25 | 0 | 22 |
| 0.6 | 26.7 | 0.4 | 22.4 |
| 1.6 | 28.6 | 1.2 | 23.7 |
| 2.6 | 30.2 | 2 | 25.1 |
| 3.7 | 31.3 | 2.8 | 26.5 |
| 4.9 | 32.2 | 3.7 | 28 |
| 5.8 | 34 | 4.9 | 30 |
| 6.9 | 34.8 | 5.7 | 32.9 |
| 7.7 | 36.3 | 6.9 | 35 |
| 8.7 | 37.4 | 7.7 | 36.4 |
| 9.6 | 38.6 | 8.6 | 37.8 |
| 10.5 | 39 | 9.8 | 40.2 |
| 11.6 | 40.5 | 10.2 | 41.3 |
| 13 | 41.3 | 11.4 | 44.4 |
| 13.5 | 42.4 | 12.7 | 46.3 |
| 14.5 | 43.9 | 13.9 | 49.9 |
| 15.9 | 45.1 | 14.7 | 52.8 |
| 16.9 | 46.2 | 15.9 | 59.4 |
| 18 | 46.4 | 16.8 | 63.1 |
| 18.6 | 49 | 17.6 | 68.1 |
| 19.9 | 50.1 | 18.4 | 72.2 |
| 20.5 | 51.2 | 19.2 | 76.8 |
| 20.9 | 51.9 | 20.5 | 84.7 |
| 21.5 | 52.5 | 20.9 | 85.7 |
| 21.8 | 54.6 | 21.7 | 88.4 |
| 22.6 | 55.4 | 22.1 | 90 |
| 22.8 | 55.8 | 22.9 | 90.4 |
| 23.1 | 56.9 | 23.3 | 91 |
| 23.5 | 57.8 | 23.7 | 91.2 |
| 23.8 | 58.8 | 24.1 | 94.7 |
| 23.9 | 60 | 24.6 | 93.9 |
| 24.2 | 60.9 | 25 | 94.7 |
| 24.4 | 62.3 | 25.4 | 95.5 |
| 24.6 | 63.4 | 26.2 | 97.2 |
| 24.8 | 64.9 | 26.6 | 98.5 |
| 25.2 | 69.1 | 27 | 99.2 |
| 25.8 | 71.4 | 27.4 | 99.9 |
| 25.9 | 73.3 | 27.8 | 101 |
| 26.1 | 77.5 | 28.2 | 101.7 |
| 26.5 | 79.1 | 28.7 | 102.4 |
| 26.6 | 80.2 | 29.5 | 104.2 |
| 26.7 | 84.4 | 29.9 | 104.9 |
| 27.1 | 87.8 | 30.3 | 106 |
| 27.5 | 89.4 | 30.7 | 106.7 |
| 27.7 | 90.9 | 31.5 | 108.6 |
| 27.9 | 92 | 31.9 | 109.6 |
| 28 | 94 | 32.3 | 110.5 |
| 28.4 | 94.3 | 32.8 | 111 |
| 28.7 | 94.4 | 33.2 | 111.7 |
| 28.9 | 96.8 | 33.6 | 112.4 |
| 29.5 | 100.5 | 34.4 | 114.5 |
| 30.5 | 102 | 34.8 | 115.8 |
| 30.7 | 102.4 | 35.2 | 117.1 |
| 30.8 | 108 | 35.6 | 120.1 |
| 31.5 | 109.2 | 36 | 120.4 |
| 32 | 110.4 | 36.4 | 121.6 |
| 32.7 | 121.3 | 37.3 | 125.3 |
| 33.4 | 135.2 | 37.7 | 127.2 |
| 33.9 | 140 | 38.1 | 128.9 |
| 34 | 147.8 | 38.9 | 130.9 |
| 34.1 | 157 | 39.3 | 132 |
| 34.2 | 163.9 | 39.7 | 133.6 |
| 34.3 | 168.4 | 40.1 | 135 |
| 34.4 | 175.3 | 40.5 | 138.4 |
| 34.5 | 180.3 | 41 | 142.7 |
| 34.6 | 187.9 | 41.1 | 147.3 |
| 34.7 | 189.4 | 41.2 | 156.8 |
| 34.8 | 194.4 | 41.3 | 161.8 |
| 34.9 | 200.5 | 41.4 | 167.6 |
| 35 | 210.4 | 41.5 | 173.2 |
| 35.1 | 230.4 | 41.6 | 187.1 |
| 35.2 | 249.7 | 41.7 | 198.8 |
| 35.3 | 275.3 | 41.8 | 210.5 |
| 35.4 | 308.2 | 41.9 | 224.2 |
| 35.45 | 338.2 | 42 | 237.1 |
|  |  | 42.1 | 259.6 |
|  |  | 42.2 | 297.3 |
|  |  | 42.3 | 360.1 |
